# Esthetic Outcomes of ADM-Assisted Expander-Implant Breast Reconstruction

**Published:** 2012-12-19

**Authors:** Khang T. Nguyen, Lauren M. Mioton, John T. Smetona, Akhil K. Seth, John Y. Kim

**Affiliations:** ^a^Northwestern University Feinberg School of Medicine, Chicago, Ill; ^b^Vanderbilt School of Medicine, Nashville, Tenn; ^c^Division of Plastic and Reconstructive Surgery, Northwestern University, Chicago, Ill

## Abstract

**Objective:** Adjunct acellular dermal matrices (ADM) are thought to improve esthetic outcomes of breast reconstruction but the existing evidence is largely anecdotal. In this study, we provide comparative data on esthetic outcomes of expander-implant breast reconstruction with and without ADM. **Methods:** Chart review was performed on a consecutive series of expander-implant reconstructions by the senior author. Demographic, oncologic, surgical, and photographic data were obtained for each patient. Photographic data were scored using a 3-point (0-1-2) breast-specific esthetic scale by 3 blinded, independent reviewers not involved in patient care. **Results:** ADM-assisted breast reconstructions had significantly higher scores than the non-ADM reconstructions for breast mound volume (1.38 vs 1.11; *P* = .0102), breast mound placement (1.57 vs 1.39; *P* = .0217), and the inframammary fold (1.39 vs 1.23; *P* = .0458). **Conclusions:** ADM may improve breast volume, placement, and inframammary fold definition. These specific findings may help plastic surgeons better utilize ADM to improve outcomes for breast reconstruction.

Acellular dermal matrices (ADMs) have become popular adjuncts for expander-implant–based breast reconstruction. Acellular dermal matrix slings are used to provide extended coverage of tissue expanders and implants inserted subpectorally, with inferolateral slings being most common.^1^ Acellular dermal matrix has been reported to support larger and faster tissue expansion, reduce capsular contracture, decrease the rate of revision, and most importantly, improve esthetic outcomes.^2^^-^14

An extensive literature review by Nguyen et al^15^ found that while there is significant evidence suggesting faster tissue expansion and reduced capsular contracture with ADM-assisted expander-implant breast reconstructions, few studies have rigorously evaluated the esthetic benefits. Instead, many reports have only provided anecdotal support for improved cosmesis.^2^^-^9 Furthermore, the small number of studies that provide comparative evidence of esthetic benefits with ADM use have relied on scoring scales that only rate the overall outcomes of reconstructions (ie, poor, fair, excellent) without specific criteria.^12^^-^14

Thus, this study provides much-needed quantitative evaluation of esthetic outcomes for expander-implant breast reconstruction with ADMs using a validated esthetic scoring scale.^16^ Importantly, 5 critical esthetic elements of the breast—breast mound volume, placement, contour, scarring, and inframammary fold definition—are scored to provide a more precise understanding of the esthetic benefits of adjunct ADMs.

## METHODS

With the approval of the institutional review board, retrospective chart review was carried out on a consecutive series of expander-implant breast reconstructions by the senior author. Demographic, oncologic, surgical, and photographic data were obtained for each patient. Demographic and oncologic variables included the following: age, body mass index (BMI), active smoking status, diagnosed diabetes, postmastectomy radiation therapy, prior history of radiation therapy, and chemotherapy. Surgical variables included reoperation due to complications or for cosmesis and symmetry. Criteria for inclusion in the analysis were completion of expander-implant exchange and photographically documented postexchange follow-up of at least 90 days.

Three blinded members of the Division of Plastic Surgery who did not participate in care of the patients were asked to independently rate postoperative anterior photographs of patients’ breasts using a 3-point scale (0-1-2) with respect to 5 distinct esthetic criteria: breast mound volume, contour, placement, scarring, and inframammary fold. As described by Lowery et al,^16^ a score of zero in each of the respective categories represented the following: marked discrepancy in volume relative to the contralateral side; marked contour deformity or shape asymmetry; marked displacement of the breast mound; hypertrophic scars and evident contracture; and a poorly defined inframammary fold. A rating of one indicated only mild differences relative to the contralateral side, fair scarring (ie, poor color match or wide scars without hypertrophy or contracture), and a defined yet asymmetrical inframammary fold. Any criteria with a score of 2 on the Lowery scale had superior esthetic outcomes—namely, minimal differences in volume, contour or placement, thin scars, and symmetrical inframammary folds. Esthetic scores of the ADM and non-ADM cohorts were compared with the Student *T* tests. Demographic variables were compared with the Student *T* test for continuous variables and chi-squared test or the Fischer exact test for dichotomous variables as appropriate. All analyses were performed with SPSS version 20.0 (IBM Corp, Armonk, New York).

## RESULTS

Of 115 patients meeting inclusion criteria, 53 had expander-implant reconstruction without adjunct ADM and 58 had expander-implant reconstruction with ADM. Characteristics of each group are shown in Table 1. Mean BMI was significantly greater in the ADM group (*P* = .0099). Age, active smoking status, diabetes, prior radiation, postmastectomy radiation, chemotherapy, and reoperations rates were similar between groups.

Esthetic scoring of breast mound volume, contour, placement, scarring, and the inframammary fold in reconstructions with and without ADM are shown in Table 2. In general, the highest scoring category for both groups of reconstructions was breast mound placement, while the lowest scoring category for both groups was breast mound contour. The ADM group had significantly higher scores than the non-ADM group for breast mound volume (1.38 vs 1.11; *P* = .0102), placement (1.57 vs 1.39; *P* = .0217), and the inframammary fold (1.39 vs 1.23; *P* = .0458).

## DISCUSSION

Acellular dermal matrices have become popular adjuncts for extending coverage of expanders and implants during breast reconstruction and augmentation. Studies have demonstrated that use of ADMs presents an acceptable increased risk of complications in exchange for a number of potential benefits, including improved esthetic outcomes.^17^^-^19 However, many of the proposed benefits of ADMs for breast reconstruction are only supported by anecdotal evidence.^15^

In the case of esthetic outcomes, a number of authors have reported positive experiences with ADMs,^2^^-^9 but few studies have methodically and quantitatively evaluated esthetic outcomes with appropriate controls.^12^^-^14 In these latter cases, the authors rely on scoring the overall esthetic appeal of the reconstructed breast(s) but often do not specifically quantify differences in contracture, prosthesis migration, lower pole expansion, and inframammary fold definition, which are thought to be the key esthetic advantages of ADMs. Here, we present a quantitative comparison of ADM-assisted and non-ADM expander-implant breast reconstruction using a validated scoring scale of 5 breast-specific esthetic elements.

### Esthetic outcomes

The ADM group had significantly higher esthetic scores for breast mound placement, volume, contour, and inframammary fold than the non-ADM group. These results are consistent with previous reports suggesting that ADM improves lower-pole expansion and definition of the inframammary fold by preventing capsular contracture and migration of expanders and implants superiorly.^3^^,^^4^^,^^6^^,^^13^ Likewise, improved volume of the breast mound may result from ADM facilitation of greater initial fill volumes of tissue expanders and larger permanent implants.^2^^,^^3^^,^^5^^,^^9^

Acellular dermal matrices did not improve the appearance of scars. Though it is thought to provide additional soft tissue coverage of prostheses and reduce tension on overlying surgical wounds, overly aggressive tissue expansion and insertion of larger implants may paradoxically lead to increased adverse effects on wound healing and scarring. Indeed, previous meta-analysis by our group suggests that ADM-assisted reconstructions experienced higher rates of tissue flap necrosis than non-ADM reconstructions.^17^ Acellular dermal matrices also did not appear to improve the overall contour of the breast mound. A number of factors may affect breast contour in the final reconstruction, including capsular contracture, shape and texture of the permanent implant, and fat injection procedures. As implant choice and fat injections were often dependent on patient preferences, these factors were likely confounders.

Interestingly, while the ADM group had significant esthetic advantages, there were no differences in reoperations for any cause (complications and cosmetic) between groups. As mentioned earlier, ADMs may reduce the rate of revisions due to prosthesis migration or capsular contracture but alternately may increase rate of revision for other complications. In terms of reoperation for cosmetic reasons, ADMs may reduce the rate of gross deformities of the reconstructed breast, but it is probable that fine adjustments to breast mound contour, volume, and position require additional procedures within the standard of care (ie, augmentation, reduction, mastopexy, fat injection). Again, patient preferences, satisfaction with reconstruction, and tolerance for additional surgery all play a role in decisions to reoperate for cosmetic defects and likely confounded the rates in this study.

### Patient characteristics

The ADM and the non-ADM groups were generally similar. Most demographic and historical factors that may have confounded esthetic outcomes, such as radiotherapy, chemotherapy, smoking, and diabetes, were not significantly different between groups. Notably, the ADM group had a greater mean BMI than the non-ADM group, which is consistent with the idea that ADM benefits obese patients with wide chest walls by improving subpectoral coverage and stabilization of large expanders and implants.^1^ However, obesity has also been traditionally associated with greater rates of complications.^20^^-^22 Thus, preferential use of ADMs in this high-risk population could be expected to diminish potential esthetic benefits in a number of ways. For example, increased rates of mastectomy flap necrosis and wound infection could increase visible scarring, while an increased rate of contracture could dramatically affect final breast mound placement, contour, and volume. Despite such potential risks, previous reports indicate satisfaction with breast reconstructions to be similar between obese and nonobese patients, suggesting that obesity may not dramatically affect final esthetic outcomes.^23^

### Study limitations

Esthetic scoring was performed on all patients initially undergoing implant-expander reconstruction with at least 90 days of photographically documented postexchange follow-up. A minimum follow-up period was used to allow perioperative complications that might transiently affect cosmesis to resolve before scoring. Patients with a complicated course potentially requiring conversion to autologous flap-based reconstruction were not excluded from the study, which could have introduced confounding variables into the analysis. However, in our single surgeon cohort, exclusion of these patients would have limited the power of the study and bias findings toward uncomplicated outcomes. Because ADM and non-ADM reconstructions experienced a similar number of revisions (not all of which involved conversion to an autologous flap), we included patients with revisions with the expectation that confounders were evenly distributed between ADM and non-ADM groups. In future work, we hope to analyze long-term follow-up data to determine whether esthetic differences persist between ADM and non-ADM cohorts.

Because of the retrospective design of the study, it is also difficult to correct for potential bias with respect to selection of patients for ADM-assisted reconstructions. In this single-surgeon cohort, patients were selected for ADM or non-ADM surgeries according to the surgeon's clinical judgment which included consideration of history of radiation to the breast, insufficient tissue coverage, and patient's desire for larger reconstructions or accelerated tissue expansion. To our knowledge, the exercise of clinical judgment in utilization of ADM remains the standard of care. It is our goal to incorporate these results and other outcomes research in future efforts to develop clinical guidelines for the use of ADMs in breast reconstruction.

Another potential issue concerns the validity and reliability of our esthetic scoring scale. Some authors have argued that computer-assisted volumetric evaluations of 3-D or 2-D photography are more consistent and precise methods of evaluating esthetic outcomes.^24^ However, scoring scales continue to present a number of advantages, including intuitively meaningful data, ease of use, and significantly lower costs. Thus, we attempted to use a validated scale with breast-specific, well-described subcriteria to characterize esthetic outcomes in a clinically significant manner.^16^ The scale used in this article has been shown to have significantly better intrarater and interrater reliability when compared with scales without specific criteria, such as visual analog scales.^16^ Furthermore, the validity of the scale has been demonstrated using multiple raters scoring postoperative photographs of breast reconstructions in a fashion similar to this study.

Finally, it is important to address the clinical significance of these results. It is our contention that the current state of esthetic outcomes research in the field of breast reconstruction has relied largely on surgeons’ subjective evaluations, which may differ significantly between surgeons and with patient views. This anecdotal evidence has often driven the development of new techniques such as the incorporation of ADMs in reconstructions. While a quantitative model may seem counterintuitive to gestalt appreciations of esthetic forms, such an approach reduces subjective biases and can be used to corroborate the purported advantages of various techniques—in this case, the use of adjunct ADM. Importantly, we hope to correlate these results with patient satisfaction scores in future work to further establish the clinical significance of the model proposed in this article.

## CONCLUSIONS

This study methodically and quantitatively evaluates the esthetic benefits of ADM adjuncts in expander-implant breast reconstruction. Acellular dermal matrices may improve breast volume, placement, and inframammary-fold definition. These specific findings may help plastic surgeons better utilize ADMs to improve outcomes for breast reconstruction.

## Figures and Tables

**Table 1:** Population characteristics

	Non-ADM	ADM	*P*
n	53	62	
Age, y	53.4 (32.0-81.0)	53.1 (31.0-72.9)	.8615
Body mass index	25.6 (17.5-50.1)	28.5 (19.3-43.3)	.0099
Active smoking	3/53 (5.6%)	4/62 (6.5%)	1.0000
Diabetes	2/53 (3.8%)	1/62 (1.6%)	.5941
Prior radiation	5/53 (9.4%)	3/62 (4.8%)	.4675
Postmastectomy radiation therapy	12/53 (22.6%)	13/62 (21.0%)	1.0000
Chemotherapy	24/53 (45.3%)	29/62 (47.8%)	1.0000
Reoperation due to complications	16/53 (30.2%)	20 (32.3%)	.8426
Reoperation for cosmesis and symmetry	25/41 (61.0%)	18/33 (54.5%)	.6396

**Table 2:** Esthetic outcomes of all reconstructions

	Non-ADM (n = 53)	ADM (n = 62)	*P*
Volume	1.11	1.38	.0102
Contour	0.92	1.11	.0621
Placement	1.39	1.57	.0217
Scarring	1.38	1.36	.8055
Inframammary fold	1.23	1.39	.0458
